# Sequential effects of reappraisal and rumination on anger during recall of an anger-provoking event

**DOI:** 10.1371/journal.pone.0209029

**Published:** 2019-01-02

**Authors:** Carmen Peuters, Elise K. Kalokerinos, Madeline Lee Pe, Peter Kuppens

**Affiliations:** 1 Faculty of Psychology and Educational Sciences, KU Leuven, Leuven, Belgium; 2 Department of Movement and Sports Sciences, Ghent University, Ghent, Belgium; 3 School of Psychology, The University of Newcastle, Callaghan, Australia; 4 European Organization for Research and Treatment of Cancer, Brussels, Belgium; University of Graz, AUSTRIA

## Abstract

In everyday life, people often combine strategies to regulate their emotions. However, to date, most research has investigated emotion regulation strategies as if they occur independently from one another. The current study aims to better understand the sequential interplay between strategies by investigating how reappraisal and rumination interact to affect anger experience. After participants (N = 156) recalled a recent anger-provoking event, they were instructed to either a) reappraise the event twice, b) reappraise the event, and then ruminate about the event, c) ruminate about the event, and then reappraise the event, or d) ruminate twice about the event. The effects of the first strategy used replicated a large body of research: reappraisal was associated with a decrease in anger, but rumination was associated with no change in anger. There was a small interactive effect of the combination of the two strategies, such that those who ruminated and then reappraised showed a larger decrease in anger than those who reappraised and then ruminated. There were no other differences between groups. This suggests that the second strategy does have an effect over and beyond the first strategy, but this effect is small in size, highlighting the importance of the initial emotion regulation strategy used.

## Introduction

Imagine you are stuck in traffic. You may feel angry. First, you ruminate, dwelling on your anger, and thinking about all the precious time you are losing. Next, you try to think of the event from a more objective perspective: in the grand scheme of things, traffic is just a minor nuisance. We regulate our emotions in a myriad of complex ways, and this scenario illustrates one such complexity: we can use multiple strategies to regulate our emotions in response to a single event.

People often spontaneously use multiple emotion regulation strategies in response to a single stimulus, both in the laboratory (e.g. [1]) and in daily life (e.g. [2,3]). However, most research examines emotion regulation strategies independently from one another, asking participants to use a single specific strategy in the lab [4], or to self-report specific strategy use using questionnaires (e.g. [5]). As a result, we know very little about how emotion regulation strategies interact, and in particular, how the order in which strategies are used may change their relationship with emotional experience. In this study, we examined how the order in which two emotion regulation strategies (rumination and reappraisal) were implemented affected anger experience across time.

### The interplay between multiple emotion regulation strategies

To date, very little research has examined how strategies are combined with each other, and even fewer studies have examined order effects in emotion regulation. To our knowledge, only two published studies have directly examined the sequential use of multiple emotion regulation strategies [6,7]. Both studies induced negative mood, and asked participants to engage in periods of rumination and distraction.

Trask and Sigmon [7] conducted two experiments in which they induced depressed mood and examined order effects of instructed rumination and distraction. They found that the initial use of rumination maintained depressed mood, and the initial use of distraction lowered depressed mood. They also found an effect of strategy order: when participants engaged in distraction following rumination, their depressed mood was reduced. When participants engaged in rumination following distraction, they did not report a change in mood. This suggests that distraction can counteract depressed mood, even when implemented after rumination. It also suggests that rumination does not reverse the benefits of initial distraction. In their second experiment, the researchers also investigated the effects of engaging in the same strategy twice. In the rumination-rumination condition, they found that there were no changes in depressed mood with the passage of time. In the distraction-distraction condition, they found initial reductions in depressed mood after the first strategy implementation, but no additional reductions with the second implementation, suggesting there were no benefits to repeated distraction.

Yoon and Joormann [6] induced sadness, and investigated the sequential effects of rumination and distraction on both negative mood and interpersonal problem solving. They found that initial engagement in distraction predicted better performance on the interpersonal problem-solving task even when it was followed by rumination, but initial engagement in rumination predicted poorer performance on the task even when it was followed by distraction. This suggested that the initial strategy was most important. However, they did not find an equivalent pattern on negative mood. After the implementation of the second emotion regulation strategy, participants in the rumination-distraction group were in a less negative mood than those in the distraction-rumination and rumination-rumination groups. There was no difference between the rumination-distraction group and the distraction-distraction group at this time-point. This is in line with the results of Trask and Sigmon [7] in suggesting that distraction can still be effective in reducing negative mood after initial rumination.

Taken together, the research suggests that changing from a putatively maladaptive strategy like rumination to a putatively adaptive strategy like distraction could help down-regulate negative mood. However, these two papers both focused on the emotion regulation strategies distraction and rumination. In addition, the studies had small sample sizes (N = 51 [6], Ns = 43 and 47 after exclusions [7]), which meant that cell sizes were small in analyses focused on four groups. Finally, these studies examined depressed mood [7] and sadness [6]. Given that switching between emotion regulation strategies occurs relatively often in daily life [3], we believe it is important to extend this body of work to a larger sample, to different emotion regulation strategies, and to other specific emotions. In this study, we focus on anger, a high arousal emotion, which represents a different approach to previous studies that focused on low arousal emotions sadness and depressed mood [8]. We chose anger to extend this work to another specific emotion, and also because rumination and reappraisal have clear and divergent effects on anger, which we will outline in the coming paragraphs.

### Rumination and reappraisal

In this study, we investigate two cognitive emotion regulation strategies: rumination and reappraisal. Rumination, as investigated in the two previous papers discussed earlier, involves repetitively and passively focusing on one’s emotions about an emotional event, or the event itself [9]. Reappraisal involves reinterpreting an emotional event by changing one’s perspective about it, allowing the regulator to take a more objective view [10]. Both strategies involve cognitive engagement with an emotion-eliciting event. However, generally rumination is seen as a maladaptive emotion regulation strategy, and reappraisal as an adaptive strategy [11].

In line with this idea, reappraisal has been associated with reduced negative emotional experience and increased positive emotional experience both in trait questionnaires [5], and in daily diary studies [12]. Reappraisal is also associated with larger short-term decreases in negative emotion than acceptance, another putatively adaptive emotion regulation strategy [13]. When used in response to recall of an anger-provoking memory, reappraisal shows an adaptive profile of cardiovascular responding [14] and is effective in down-regulating anger experience [15,16].

In contrast, rumination is associated with greater negative affect in both cross-sectional and experimental studies [17], as well as in experience sampling studies [2]. Relative to reappraisal, rumination leads to greater anger experience and more repetitive thinking about an angering event [16,18–21]. Rumination is also associated with greater negative affect than reappraisal in response to a sad memory [22].

We chose to investigate rumination and reappraisal for two reasons. First, they are both cognitive emotion regulation strategies that involve engaging with thoughts about an event [22]. Thus, they may be two sides of the same coin: a person could switch between ruminating on an event and reappraising it quickly, without changing their focus of attention. This is in contrast to other common emotion regulation strategies like distraction and suppression, which involve directing attention away from an emotional event.

Second, like distraction, reappraisal is associated with reduced negative emotion [19], meaning that both are potentially effective counters to rumination in response to angry events. However, reappraisal is more cognitively burdensome than distraction [23,24], and likely for this reason, distraction tends to be chosen over reappraisal when targeting high-intensity stimuli [25]. Indeed, models of temporal emotion regulation suggest that because reappraisal targets later processing stages, it is likely to be less effective when emotions are at high intensity. This is in contrast to distraction, which targets earlier processing stages, and will thus be relatively unaffected by emotional intensity [26,27]. As we outline in the coming paragraphs, these differences between reappraisal and distraction mean that we may find differences between our study and the previous research focused on distraction.

### The order of reappraisal and rumination

As we noted above, rumination is generally seen as a maladaptive emotion regulation strategy. However, increasingly, researchers have emphasized the importance of the context in which emotion regulation occurs in determining whether a strategy is adaptive [28]. In the current study, the context in which the second strategy is occurring is set by the first strategy. We investigate the effects of reappraisal followed by rumination, and rumination followed by reappraisal. We also investigate the effects of using each strategy consecutively, that is, reappraisal followed by reappraisal, and rumination followed by rumination. Below, we discuss each of these four conditions.

#### Reappraisal followed by rumination

When deploying rumination following reappraisal, the participant is beginning to ruminate on the event in a context where they have already generated reappraisals that have changed their understanding of the event. Research has demonstrated that rumination only leads to negative affect if what is being ruminated on is negative [29]. Thus, those who reappraised before ruminating may be buffered against subsequent negative emotional experience because the thoughts they are ruminating on have already been made less negative by the reappraisal. This suggestion is supported by research conducted by vanOyen Witvliet and colleagues [16]. They found that when participants initially used compassionate reappraisal in response to a past interpersonal offence, when later ruminating, they showed greater empathy towards the offender. This suggests that the context and content of rumination was changed by the initial use of reappraisal. In line with this study, we suggest that when following reappraisal, rumination may be associated with a greater reduction in negative affect than when it follows rumination.

#### Rumination followed by reappraisal

People report finding reappraisal a difficult emotion regulation strategy to implement [13,30], and psychophysiological data suggests that implementing reappraisal may be cognitively taxing [16,23,24]. This difficulty may be compounded by the fact that initial rumination is likely to make negative thoughts highly accessible [31,32], and to reduce concentration [33]. Thus, initial rumination might impede the effective implementation of later reappraisal. Therefore, when following rumination, reappraisal may be associated with a smaller reduction in negative affect than when it follows reappraisal.

#### Staying with the same strategy: Rumination followed by rumination, and reappraisal followed by reappraisal

In our study, we also include conditions in which participants repeat the same emotion regulation strategy twice. This is important in order to establish the natural trajectories of these strategies. Additionally, an interesting aspect of these conditions is that they allow us to test whether continued rumination is associated with continued maintenance of negative emotion, and continued reappraisal is associated with additional reductions in negative emotion. In the second study conducted by Trask and Sigmon [7], continued rumination was associated with continued maintenance of negative emotion, but continued distraction was not associated with additional reductions in negative emotion. In our study, we will determine whether this is also the case for reappraisal.

### The current study

To investigate the interplay between reappraisal and rumination, we based our study on a paradigm used by Ray et al. [18]. To test the effects of strategy type and order on anger experiences, we asked participants to recall their most recent angering event. We then asked participants to: a) reappraise the event twice, b) ruminate about the event twice, c) reappraise the event first and then ruminate, or d) ruminate about the event first and then reappraise. We focus on anger experience as our key dependent variable, since our manipulation targeted anger in particular, rather than negative affect more generally.

We had two sets of hypotheses. First, because reappraisal allows individuals to take a more objective view on their emotions [10] and in line with a large body of research [4], we hypothesized that the use of reappraisal would decrease anger. In contrast, because rumination leads to perseverative negative thinking [18] and also in line with a large body of research [4,34], we hypothesized that the use of rumination would maintain levels of anger (*Hypothesis 1)*.

Second, we hypothesized that the order in which the strategies were implemented would affect their impact on anger experiences. Here, we had three specific hypotheses. Because initial reappraisal could change thoughts about the event in a way that buffered against rumination, we hypothesized that after the implementation of rumination as a second emotion regulation strategy, those that had previously reappraised (the reappraisal-rumination condition) would show greater decreases in anger than those that had previously ruminated (the rumination-rumination condition; *Hypothesis 2a)*. Because initial rumination may make it difficult to implement later reappraisal, we hypothesized that after the implementation of reappraisal as a second emotion regulation strategy, those that had previously ruminated (the rumination-reappraisal condition) would show greater decreases in anger than those that had previously reappraised (the reappraisal-reappraisal condition; *Hypothesis 2b*). Finally, based on the existing research on combining emotion regulation strategies [6,7], we hypothesized that those who switch from a less effective strategy to a more effective strategy (i.e. from rumination to reappraisal) would show greater decreases in anger than those who switched from a more effective strategy to a less effective strategy (i.e. from reappraisal to rumination; *Hypothesis 2c*).

## Materials and methods

### Participants

Participants were 158 Belgian first-year psychology students who participated in return for partial course credit. We excluded the data of two participants who did not comply with event recall instructions, leaving a final sample of 156 participants (132 women; *M*_age_ = 18.55, *SD*_age_ = 2.26, age range: 16–38).

### Design

We used a 2 (initial emotion regulation strategy: reappraisal vs. rumination) x 2 (second emotion regulation strategy: reappraisal vs. rumination) between-subjects design, giving us four different emotion regulation conditions (rumination-rumination, rumination-reappraisal, reappraisal-rumination, reappraisal-reappraisal). We assessed emotions within-subjects at six different time-points. Our key dependent variable was anger experience.

### Procedure

We delivered all instructions on a computer. The verbatim instructions received by participant are available at the Open Science Framework: https://osf.io/tsy3d/.This project was ethically approved by the KU Leuven Social and Societal Ethics Committee. Before beginning the experiment, participant provided written informed consent to participate. They were told that they would be participating in a study investigating the relationship between cognition and emotion.

**Fig 1:**
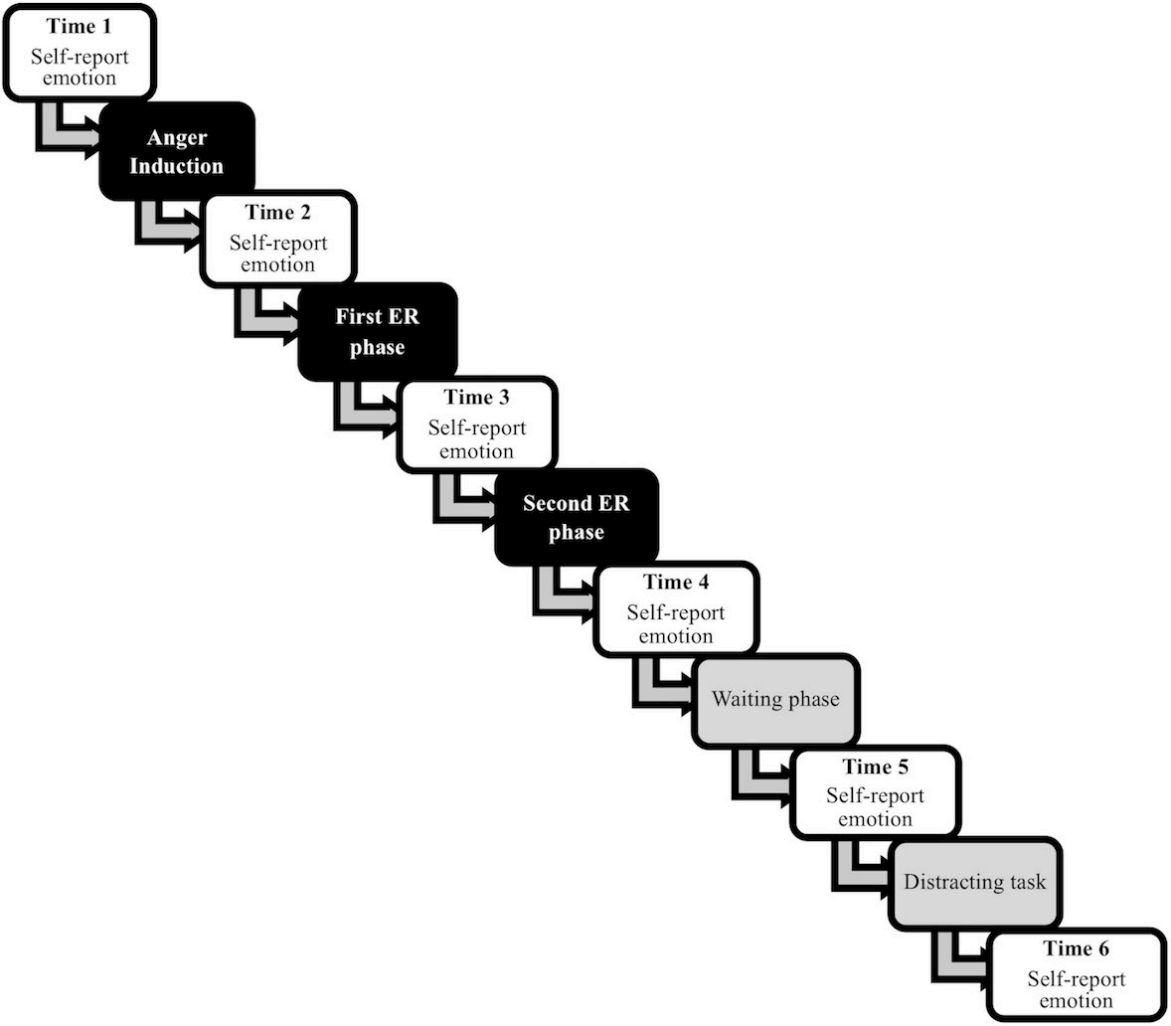
Overview of the experimental procedure, highlighting each time-point that emotion was measured. ER = emotion regulation.

An overview of the experimental procedure is presented in Fig 1. First, we asked participants to self-report their *baseline* emotions (Time 1). Next, we instructed participants to *recall a recent angering event* that had made them feel angry with another person and had not yet been fully resolved. We asked them to write a brief description of this event. Then, we asked participants to recall the event as if it were happening right now for one minute, and then to self-report their current emotions (Time 2).

Subsequently, we randomly assigned participants to one of four different conditions: (a) ‘*reappraisal-reappraisal*’ condition (N = 41), in which participants were instructed to first reappraise for one minute, and then reappraise for another minute; (b) ‘*rumination-rumination*’ condition (N = 40), in which participants were instructed to first ruminate for one minute, and then ruminate for another minute; (c) ‘*reappraisal-rumination*’ condition (N = 34), in which participants were instructed to first reappraise for one minute, and then ruminate for another minute; and (d) ‘*rumination-reappraisal*’ condition (N = 41), in which participants were instructed to first ruminate for one minute, and then reappraise for another minute.

The emotion regulation instructions were adapted from Ray et al., 2008 [17]. The reappraisal instructions were: “*Think about the angering event from the perspective of an outsider (someone that has the best intentions for all persons involved)*. *Concentrate especially on how this person might see the event and the positives that he or she could find in it”*. To help participants reappraise, we added guide questions to the screen (e.g., “How would this person see the event?”, “What positive outcomes of the event could this person think of?”). These questions were intended to give participants assistance in using the strategy over the 1-minute regulation period, and to help avoid participants spontaneously switching to other regulation strategies.

The rumination instructions were: “*Think about the angering event from your own perspective*. *Concentrate especially on the things that originally triggered the emotions and your reactions*.”. As with the reappraisal condition, to help participants ruminate, we also added guide questions (e.g., “Why were you angry?”, “What made you feel this way?”).

We assessed current emotions following the first emotion regulation phase (Time 3) and the second emotion regulation phase (Time 4). After these two emotion regulation phases, we asked all participants to wait while looking at a picture of a ball of yarn at the screen for 30 seconds, after which we asked them to again self-report their emotions (Time 5). The purpose of including the waiting period was to examine how emotion ratings would evolve over time.

Finally, to help remove the effects of our manipulations, we asked participants to respond to a distracting task and self-report their emotions one last time (Time 6). For the distraction task, participants were instructed to press a different key for each time “*” versus “**” appeared in the same location as one of two random words that were briefly presented on the screen.

We also assessed some other variables at Time 2, 3, 4, and 5. First, we assessed participants’ emotional appraisals of the event: participants self-reported their perceived control and responsibility in the event, and the intensity and importance of the event. They also reported the time they spent thinking about the event, and the effort they expended thinking about the event. Because these ratings were not applicable to our a priori hypotheses about the research questions at hand, they were omitted from this study’s analyses.

### Measures

#### Momentary anger, positive affect and non-anger related negative affect

At each of the six time-points, participants were asked to rate several emotions on a 7-point scale (0 = not at all, 6 = very much). The questions appeared in a random order. We created a composite measure of anger, our key dependent variable, by averaging anger, fury, and irritation (between-person reliability R_KF_ = .96, within-person reliability R_C_ = .77). We also created a composite measure of non-anger related negative affect (non-anger NA) by averaging sadness, anxiety, and guilt (R_KF_ = .96, R_C_ = .51), and a composite measure of positive affect (PA) by averaging happiness, hopefulness, love and relaxation (R_KF_ = .98, R_C_ = .67). The two reliability coefficients presented here are for multilevel data, and are taken from Shrout and Lane, 2012: R_KF_ is a between-person reliability coefficient, estimating the reliability as an average over k time points for fixed coefficients (F), and is indicative of the consistency of item responses over time and across people. R_C_ is a within-person reliability coefficient, estimating the reliability of change within people over time, and is indicative of consistency between items within individuals.

## Results

The dataset and code used in this study is publicly available via the Open Science Framework, and can be accessed using the following link: osf.io/tsy3d.

Table 1 outlines the mean and standard deviations of anger, NA, and PA at each time-point. Given we induced anger, our key dependent variable was anger experience, but we report results with non-anger related NA and PA in the Supporting Information (S1 Tables: non-anger NA, S2 Tables: PA).

**Table 1:** Means of self-reported anger about the angering event for each condition, with standard deviations in parentheses.

Time	Condition			
	Rumination-Rumination	Rumination-Reappraisal	Reappraisal-Rumination	Reappraisal-Reappraisal
**1** **baseline**	0.77 (0.70)	1.08 (1.12)	0.87 (0.70)	1.09 (0.80)
**2** **post anger induction**	2.69 (1.61)	3.05 (1.67)	3.25 (1.39)	3.46 (1.37)
**3** **post first ER phase**	2.63 (1.52)	3.16 (1.66)	2.31 (1.55)	2.50 (1.23)
**4** **post second ER phase**	2.67 (1.56)	2.79 (1.63)	2.58 (1.56)	2.44 (1.39)
**5** **post waiting phase**	2.11 (1.43)	2.19 (1.54)	1.65 (1.43)	1.90 (1.46)
**6** **post distracting task**	1.58 (1.39)	1.50 (1.27)	1.53 (1.66)	1.65 (1.27)

Anger = anger ratings; ER = emotion regulation.

**Fig 2:**
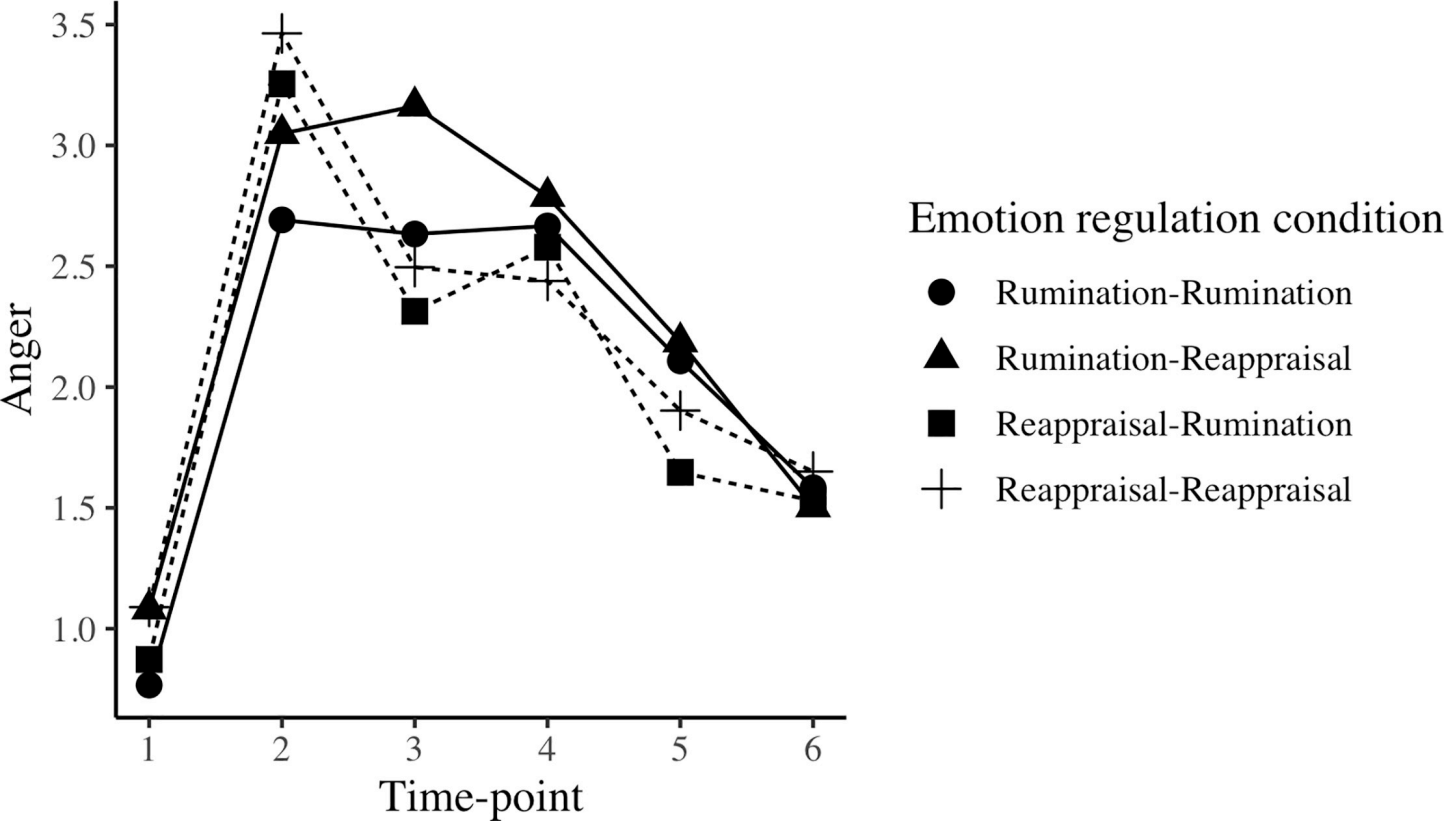
The trajectories of anger for each of the four conditions across the six time-points. The anger induction is implemented before Time 2, the first emotion regulation strategy before Time 3, and the second emotion regulation strategy before Time 4. Participants completed a waiting phase (30 seconds) between Time 4 and Time 5, and a distracting task intended to remove differences between conditions between Time 5 and Time 6. Conditions that ruminated first are represented with a solid line, and conditions that reappraised first are represented with a dashed line. ER = emotion regulation.

Fig 2 depicts the anger trajectories for each condition across the six time-points. The data are multilevel, with time-points (1 through 6) nested within subjects. To analyze the data, we used the lme4 package in R [35] to fit linear mixed effects model to the data. We calculated p-values using the lmerTest package in R, which uses a Satterthwaite approximation for degrees of freedom [36]. Given that the intervals between time assessments were not equal, we modelled time as a categorical variable. This approach allowed us to focus on differences between specific time-points at which the manipulations were implemented. In all models, we included random intercepts, but not random slopes, as it would not be possible to model random slopes for all the contrasts required as part of the categorical time variable. The R code for these analyses is available on OSF.

### Manipulation check

First, to check the efficacy of the anger induction (which was implemented prior to Time 2) in increasing anger, we ran a model using the categorical time variable to predict the anger scale. We set Time 1 (the only time-point before to the anger induction) as the reference category, which allowed us to compare each of the time-points after the anger induction to Time 1. The results of these analyses are in Table 2. These results demonstrate that the manipulation was successful: at every time-point, anger is significantly higher than at Time 1.

**Table 2:** Comparing anger at Time 1 to anger at each of the following time-points.

	γ	SE	*p*
**Intercept**	0.96	0.11	< .001
**Time 2**	2.15	0.11	< .001
**Time 3**	1.71	0.11	< .001
**Time 4**	1.66	0.11	< .001
**Time 5**	1.02	0.11	< .001
**Time 6**	0.61	0.11	< .001

Time 1 (prior to the anger induction) is the reference category in these analyses, and each time-point is being compared to Time 1.

### Effects of the first reappraisal and rumination manipulation

Next, we tested Hypothesis 1, that the use of reappraisal would decrease levels of anger, and the use of rumination would maintain or increase anger. For this hypothesis, we only assessed the first emotion regulation strategy used. The second strategy would not be a clear test of this hypothesis, given that the effect of the second strategies may have been impacted by the first strategy used.

We ran a model including the categorical time variable, the first emotion regulation strategy (rumination coded as 0, reappraisal coded as 1), and the interaction between time and emotion regulation strategy. We set the reference category to Time 2 (prior to the implementation of the first emotion regulation strategy), in order to determine whether there was a difference between emotion regulation conditions in the change in anger from Time 2 to Time 3 (post strategy implementation). We found a significant interaction between time and emotion regulation strategy (γ = -0.98, SE = 0.22, *p* = .001). This interaction was in line with Hypothesis 1. In the rumination group, there was no difference in anger between Time 2 (*M* = 2.87) and Time 3 (*M* = 2.90; γ = 0.03, SE = 0.15, *p* = .852). That is, those participants who ruminated maintained their anger across time. In the reappraisal group, we found that there was a significant decrease in anger between Time 2 (*M* = 3.37) and Time 3 (*M* = 2.41; γ = -0.96, SE = 0.16, *p* < .001). That is, those participants who reappraised showed a reduction in their anger across time.

Unexpectedly, when conducting these analyses, we noticed that the reappraisal and rumination groups significantly differed in anger at Time 2, *before* the emotion regulation manipulation had been implemented (γ = 0.50, SE = 0.22, *p* = .027). This small pre-existing difference suggested a failure of random assignment. However, importantly, this pre-existing difference was in the direction opposite to our predictions: people in the rumination condition felt significantly *less* anger prior to the rumination manipulation (*M* = 2.87) than people in the reappraisal condition (*M* = 3.37). This difference made the sample a conservative test of Hypothesis 1, which was supported despite these countervailing pre-existing differences. In addition, our key tests focus on within-group differences across time, rather than between-group differences, which means that this pre-existing difference should not affect our analyses.

### Influences of strategy order

Next, we tested Hypotheses 2a, 2b, and 2c, which concerned strategy combination effects. To test these hypotheses, we ran models including the categorical time variable, emotion regulation condition (with four levels: rumination-rumination, rumination-reappraisal, reappraisal-rumination, and reappraisal-reappraisal), and the interaction between time and regulation condition. All time-points were included in these analyses, but below we only highlight the comparisons between time-points critical to Hypothesis 2. In order to extract the necessary comparisons between the four emotion regulation conditions at each time-point, we ran the same model multiple times, changing the reference categories for emotion regulation condition and time. Table 3 outlines how each of the emotion regulation groups changed in anger ratings across time. Table 4 outlines the simple effects for the tests of differences between conditions across time. These analyses allow us to determine if change across time was different for the conditions and constitute a more direct test of our hypotheses. Importantly, these analyses focus on comparing the size of the change across time within groups, rather than differences between groups at each time-point. This means that differences in the mean levels of anger between groups at Time 3 are not problematic for our analyses: we are only interested in how much that anger decreases. Our key interest was in the difference between Time 3 and Time 4 (i.e. pre- and post- the implementation of the second emotion regulation strategy).

**Table 3:** Change in anger ratings between the time-points for each of the emotion regulation conditions.

	Change from Time 2 to Time 3			Change from Time 3 to Time 4			Change from Time 4 to Time 5			Change from Time 5 to Time 6		
	γ	SE	*p*	γ	SE	*p*	γ	SE	*p*	γ	SE	*p*
**Rumination-Rumination**	-0.05	0.22	.790	0.03	0.22	.879	-0.56	0.22	.011	-0.53	0.22	.017
**Rumination-Reappraisal**	0.11	0.22	.599	-0.37	0.22	.085	-0.60	0.22	.006	-0.68	0.22	.002
**Reappraisal-Rumination**	-0.94	0.24	< .001	0.26	0.24	.266	-0.93	0.24	< .001	-0.12	0.24	.621
**Reappraisal-Reappraisal**	-0.97	0.22	< .001	-0.06	0.22	.792	-0.54	0.22	.013	-0.25	0.22	.245

Significant effects at *p* > .05 are shaded in grey.

**Table 4:** Tests of differences between conditions in the size of the change in anger ratings across time-points.

	Change from Time 2 to Time 3			Change from Time 3 to Time 4			Change from Time 4 to Time 5			Change from Time 5 to Time 6		
	γ	SE	*p*	γ	SE	*p*	γ	SE	*p*	γ	SE	*p*
**Rum-Rum vs. Rum-Reap**	0.17	0.31	.446	-0.41	0.31	.187	-0.04	0.31	.888	-0.16	0.31	.609
**Rum-Rum vs. Reap-Rum**	-0.88	0.32	.007	0.23	0.32	.475	-0.37	0.32	.249	-0.41	0.32	.208
**Rum-Rum vs. Reap-Reap**	-0.91	0.31	.003	-0.09	0.31	.770	0.02	0.31	.944	0.27	0.31	.376
**Rum-Reap vs. Reap-Rum**	-1.06	0.32	.001	0.64	0.32	.047	-0.33	0.32	.306	0.57	0.32	.079
**Rum-Reap vs. Reap-Reap**	-1.08	0.31	< .001	0.32	0.31	.301	0.07	0.31	.832	0.43	0.31	.160
**Reap-Rum vs. Reap-Reap**	-0.03	0.32	.935	-0.32	0.32	.318	0.39	0.32	.220	-0.13	0.32	.676

Significant effects at *p* > .05 are shaded in grey.

Rum-Rum = Rumination–Rumination condition, Rum-Reap = Rumination–Reappraisal condition, Reap-Rum = Reappraisal—Rumination condition, Reap-Reap = Reappraisal–Reappraisal condition.

The changes between Time 2 and 3 in Tables 3 and 4 confirm the analyses we conducted to test Hypothesis 1: All groups that ruminated first showed significantly higher anger than groups that reappraised first. These differences were such that the two reappraisal groups showed a significant decrease in anger, but the two rumination groups did not show any change across time.

#### Hypothesis 2a

We hypothesized that the reappraisal-rumination condition would show a greater reduction in anger from Time 3 to Time 4 than the rumination-rumination condition. As indicated in Table 4, this hypothesis was not supported (*p* = .475).

#### Hypothesis 2b

We hypothesized that the rumination-reappraisal condition would show a smaller reduction in anger from Time 3 to Time 4 than the reappraisal-reappraisal condition. As indicated in Table 4, this hypothesis was also not supported (*p* = .318).

#### Hypothesis 2c

We hypothesized that the rumination-reappraisal condition would show a larger reduction in anger from Time 3 to Time 4 than the reappraisal-rumination condition. As indicated in Table 4, this hypothesis was supported *(p* = .047). However, we note that this effect is small in size, and close to our statistical significance threshold (*p* = .05). Referring back to Table 3, the Rumination-Reappraisal condition showed a non-significant decrease in anger, but the Reappraisal-Rumination showed no change in anger.

#### Other results

Table 4 demonstrates that there were no differences between groups in the change from Time 4 to Time 5, or Time 5 to Time 6. As outlined in Table 3, all groups showed a significant decrease in anger from Time 4 to Time 5. Only the Rumination-Rumination and the Rumination-Reappraisal groups showed a significant decrease in anger following the distracting task implemented at Time 6. This may have been because these two conditions reported higher anger at Time 5 than the other two groups, and thus anger means in these two groups could decrease more strongly after the distraction task.

## Discussion

In this study, we asked participants to recall an angering event, and investigated the impact of reappraisal, rumination, and the sequential interplay between the two strategies on self-reported anger. Supporting Hypothesis 1, and in line with a large body of existing work [18–20] we found that levels of anger decreased following initial reappraisal, but not following initial rumination.

We also had three hypotheses about strategy order effects. In Hypothesis 2a, we predicted that because initial reappraisal could reshape thoughts of an event in a way that reduces the negativity of rumination, those that ruminated after reappraising would show a greater decrease in anger than those that ruminated after ruminating. Our results did not support this hypothesis: we found no difference in the change in anger across time in the Rumination-Rumination and Reappraisal-Rumination conditions. This suggests that, despite the cognitive reshaping of the event started by the initial reappraisal, subsequent rumination inhibits negative emotion reduction. However, participants only engaged in the initial reappraisal for one minute. Perhaps with a longer initial period of reappraisal, a stronger new perspective on the event would be established, and this would better counteract ensuing rumination.

We also found that there was no change between Time 3 and Time 4 for the Reappraisal-Reappraisal group. This suggests that, despite reappraisal initially being associated with anger reduction between Times 2 and 3, continued reappraisal may not be associated with additional benefits. This result is in line with the findings of Trask and Sigmon [7] for distraction.

In Hypothesis 2b, we predicted that because initial rumination could maintain negative emotion and reduce cognitive resources, those that reappraised after ruminating would show a smaller decrease in anger than those that reappraised after reappraising. Our results also did not support this hypothesis: we found no difference in the change in anger across time in the Reappraisal-Reappraisal and Rumination-Reappraisal conditions. However, research has suggested that reappraisal may be particularly difficult in high-intensity negative events [23], and our lab recall paradigm induced only moderate negative emotion. Thus, reappraisal following rumination may have been less difficult than we anticipated. In future research, it would be interesting to investigate this hypothesis in a more emotionally intense paradigm. In daily life, the benefits of reappraisal are most apparent in the presence of rumination [37], suggesting that perhaps this combination of strategies could be implemented in a helpful way in response to daily hassles.

Finally, in Hypothesis 2c, we predicted that switching from rumination, a strategy generally less effective in regulating negative emotion, to reappraisal, a strategy generally effective in regulating negative emotion, would be more effective than the opposite pattern. Our results supported this hypothesis: we found a small effect such that the Rumination-Reappraisal condition showed a greater reduction in anger across time than the Reappraisal-Rumination condition. This result is in line with previous studies investigating the combination of rumination and distraction in regulating negative mood, which found that switching from rumination to distraction was associated with a reduction in negative mood [6,7].

As we discussed in the Introduction, reappraisal and distraction have different mechanisms of action, but both are generally effective in reducing negative emotional experience (across negative emotions) in short lab paradigms [4]. Thus, one way to integrate our results with previous research on distraction and other negative emotions is to hypothesize that this pattern may apply to strategies effective in reducing negative emotion more broadly, rather than being strategy specific. However, it may also be that there are strategy-specific or emotion-specific explanations for these similar patterns across studies. This highlights the importance of including measures of mechanisms in future work, and including a broader variety of strategies.

In sum, our results indicate that the effects of the first emotion regulation strategy used were robust. However, when considering the second strategy used, there was only one small effect of strategy order. The benefits of switching strategy from rumination to reappraisal were much less robust than the gains from using reappraisal as an initial strategy. We believe that this suggests that the initial emotion regulation choice may matter more than the subsequent strategy used, at least in short lab paradigms.

### Emotion regulation blunting

We posit that the initial strategy is most important because it dampens the impact of subsequent strategies. This effect may be similar to *emotion blunting*, in which the experience of a previous emotion blunts the experience of a subsequent emotion (e.g. [23]). This blunting effect occurs when participants experience sequential emotions that are of the opposite valence: for example, an initial negative mood can blunt a subsequent positive mood manipulation [38]. Our study could be an example of this effect with emotion regulation strategies: reappraisal and rumination have divergent effects on anger experience, and so the use of one of these strategies blunts the effect of the subsequent contrasting strategy.

Of course, the present study focused only on reappraisal and rumination. In future, it would be interesting to examine the combined use of multiple adaptive strategies. Previous research has demonstrated that experiencing similarly valenced emotions consecutively results in a process of emotion augmentation, in which the initial emotion strengthens the following emotion (e.g. [23]). This suggests the initial use of an adaptive emotion regulation strategy might enhance the effect of a subsequent adaptive emotion regulation strategy, resulting in more favorable outcomes.

### Limitations and future directions

This study is among the first to investigate the interplay between emotion regulation strategies, but it has some limitations that point to important future research directions. First, our study did not include a control condition, so we were unable to truly probe the direction of our effects independently of the natural emotional processes at work. However, previous work suggests that reappraisal reduces negative emotion, but that anger experiences in an uninstructed free recall are not significantly different from ruminating [18]. Future work including such an uninstructed control condition would provide stronger evidence for the processes at work in our research.

Second, we believe that there is much scope for future research understanding the role of strategy timing. In our study, the emotion regulation manipulation occurred shortly after the emotion induction. We made this decision to avoid the potential impact of participants’ decisions to spontaneously engage in their own choice of emotion regulation strategies (cf. [25,26]), given that such spontaneous strategies could influence the sequential order of emotion regulation. However, it may be that the impact of strategy order is different later in the emotion generation process. Indeed, research has demonstrated that the positive relationship between rumination and negative emotion is stronger later in emotional episodes, and the negative relationship between reappraisal and negative emotion is stronger earlier in emotional episodes [3], suggesting that strategy timing could play a role in the strength of the strategies’ effects. It will be important for future research to combine questions of strategy order, timing, duration, and intensity to paint a clearer picture of the complexities of emotion regulation.

In addition, the effects were small, and a more powerful replication is necessary to establish their robustness. Such a replication could also work to improve our paradigm. We investigated a recalled angering event using a lab paradigm, and thus, mean levels of anger experience were mild to moderate. It will be important to investigate strategy order effects in more emotionally intense paradigms, where recovery may prove more difficult, and thus a later strategy change may have a stronger effect. This would also allow the inclusion of more time-points to take a closer look at the emotional recovery trajectory. It will also be important to extend these sequential paradigms to other emotions, including positive emotions. This may be particularly interesting for reappraisal, as in some studies, reappraisal is associated with increased positive emotion but not decreased negative emotion [39].

In our study, we did not include any items checking whether participants adhered to the instructed emotion regulation strategy. Previous research has suggested that participants use different tactics to enact reappraisal [40], so in an attempt to keep tactics uniform across strategies, we provided guided questions to direct emotion regulation. We also kept manipulations relatively short (one minute) in attempt to reduce switching between strategies, since such switches would be problematic for examining sequential effects. However, studies have suggested that some participants do not adhere to emotion regulation instructions from the beginning of a manipulation [41]. Thus, it will important to extend studies on sequential effects in emotion regulation to include better measures of manipulation (non)-compliance. We should also note that in this study, the majority of our participants were young female students, and this may limit the generalizability of our results to other samples.

Future research should also endeavor to build a deeper understanding of the way in which emotion regulation strategies are combined. In this study, we discuss “combined emotion regulation” as multiple emotion regulation strategies that are implemented sequentially. However, it might be the case that different strategies act simultaneously [1]. This simultaneous usage seems unlikely when looking at strategies like reappraisal and rumination, but it might be feasible with strategies that target different steps of the emotion regulation process, for example, reappraisal and expressive suppression.

Finally, future research should delve deeper into the role of specific emotions and emotional arousal in these results. Previous work on strategy combination focused on low arousal negative emotions (sadness and depressed mood; [8]). The current study focused on anger, a high arousal negative emotion, and we found similar effects to the work with low arousal negative emotions. This suggests that emotional arousal may not moderate the effect of strategy combination on emotional experience, but this remains to be directly tested in future work. Such work would benefit from extending beyond emotional experience to incorporate psychophysiological measures.

### Conclusion

In conclusion, we believe that our study points to the importance of the initial emotion regulation strategy in the emotion regulation processes. Our study also demonstrates that switching from a strategy that is less effective in reducing anger experience (rumination) to a strategy that is more effective in reducing anger experience (reappraisal) later in the emotional episode may also have emotion regulatory benefits. Overall, our research highlights the importance of considering the temporal order of emotion regulation strategies in understanding effective emotion regulation.

## Supporting information

S1 Tables Analyses with non-anger related negative affect. This appendix is a replication of the analyses in the paper with non-anger related negative affect as the dependent variable.
(DOCX)

S2 Tables Analyses with positive affect. This appendix is a replication of the analyses in the paper with positive affect as the dependent variable.
(DOCX)
