# Supplementary material for: Sequential effects of reappraisal and rumination on anger during recall of an anger-provoking event
Source: PLoS One. 2019 Jan 2;14(1):e0209029. doi: 10.1371/journal.pone.0209029 (PMC6314601; doi:10.1371/journal.pone.0209029)
Supplement: S1 Tables — This appendix is a replication of the analyses in the paper with non-anger related negative affect as the dependent variable. (DOCX) [file pone.0209029.s001.docx]

# S1 Tables: Analyses with non-anger related negative affect

Table S1a outlines descriptive statistics for non-anger related negative affect.

**Table S1a. Means of self-reported negative affect about the angering event for each condition, with standard deviations in parentheses**

| **Time** | **Condition** | | | |
| --- | --- | --- | --- | --- |
|  | **Rumination-Rumination** | **Rumination-Reappraisal** | **Reappraisal-Rumination** | **Reappraisal-Reappraisal** |
| **1  baseline** | 0.76 (0.91) | 1.30 (1.27) | 0.75 (0.89) | 0.88 (0.81) |
| **2  post anger induction** | 1.41 (1.14) | 2.02 (1.39) | 1.53 (1.12) | 1.65 (1.11) |
| **3  post first ER phase** | 1.21 (1.17) | 2.21 (1.51) | 1.01 (0.95) | 1.53 (1.17) |
| **4  post second ER phase** | 1.20 (1.11) | 1.84 (1.33) | 1.06 (1.06) | 1.40 (1.10) |
| **5  post waiting phase** | 0.78 (1.00) | 1.56 (1.35) | 0.67 (0.93) | 0.98 (1.03) |
| **6  post distrac-ting task** | 0.55 (0.95) | 1.11 (1.07) | 0.64 (1.00) | 0.56 (0.75) |

NA = non-anger related negative affect ratings; PA = positive affect ratings, ER = emotion regulation.

## These analyses are exact replications of those included in the paper, but with non-anger related negative affect (non-anger NA) the dependent variable instead of anger. Please refer to the paper for a full explanation of how these analyses were conducted

## Effect of the anger manipulation

## The results of the analyses are in Table S1b, and reveal that the manipulation also impacted non-anger NA.

**Table S1b. Comparing non-anger NA at Time 1 to non-anger NA at each of the following time-points.**

|  | **γ** | **SE** | ***p*** |
| --- | --- | --- | --- |
| **Intercept** | 0.93 | 0.09 | <.001 |
| **Time 2** | 0.73 | 0.07 | <.001 |
| **Time 3** | 0.58 | 0.07 | <.001 |
| **Time 4** | 0.46 | 0.07 | <.001 |
| **Time 5** | 0.08 | 0.07 | .288 |
| **Time 6** | -0.21 | 0.07 | <.004 |

Time 1 (prior to the anger induction) is the reference category in these analyses, and each time-point is being compared to Time 1.

**Effects of the first reappraisal and rumination manipulation**

We found a significant interaction between Time 2 and 3 and emotion regulation strategy (γ = -0.30, SE = 0.15, *p* = .043). In the rumination group, there was no difference in non-anger NA between Time 2 (*M* = 1.72) and Time 3 (*M* = 1.72; γ < 0.00, SE = 0.10, *p* = 1.00). In the reappraisal group, we found that there was a significant decrease in non-anger NA between Time 2 (*M* = 1.60) and Time 3 (*M* = 1.29; γ = -0.30, SE = 0.11, *p* =.005).

**Influences of strategy order**

Table S1c outlines how each of the emotion regulation strategy groups changed across time. Table S1d outlines the simple effects for the tests of differences between conditions across time.

**Table S1c. Change between the time-points on non-anger NA for each of the emotion regulation conditions**

|  | **Change from Time 2 to Time 3** | | | **Change from Time 3 to Time 4** | | | **Change from Time 4 to Time 5** | | | **Change from Time 5 to Time 6** | | |
| --- | --- | --- | --- | --- | --- | --- | --- | --- | --- | --- | --- | --- |
|  | γ | SE | *p* | γ | SE | *p* | γ | SE | *p* | γ | SE | *p* |
| **Rumination-Rumination** | -0.20 | 0.15 | .171 | -0.01 | 0.15 | .955 | -0.43 | 0.15 | .004 | -0.23 | 0.15 | .124 |
| **Rumination-Reappraisal** | 0.20 | 0.14 | .177 | -0.37 | 0.14 | .010 | -0.28 | 0.14 | .056 | -0.46 | 0.14 | .002 |
| **Reappraisal-Rumination** | -0.52 | 0.16 | .001 | 0.05 | 0.16 | .757 | -0.39 | 0.16 | .014 | -0.03 | 0.16 | .853 |
| **Reappraisal-Reappraisal** | -0.12 | 0.14 | .398 | -0.13 | 0.14 | .368 | -0.42 | 0.14 | .003 | -0.41 | 0.14 | .004 |

Significant effects at *p* > .05 are shaded in grey.

**Table S1d. Tests of differences between conditions in the size of the change in non-anger NA across time-points.**

|  | **Change from Time 2 to Time 3** | | | **Change from Time 3 to Time 4** | | | **Change from Time 4 to Time 5** | | | **Change from Time 5 to Time 6** | | |
| --- | --- | --- | --- | --- | --- | --- | --- | --- | --- | --- | --- | --- |
|  | γ | SE | *p* | γ | SE | *p* | γ | SE | *p* | γ | SE | *p* |
| **Rum-Rum vs. Rum-Reap** | 0.40 | 0.21 | .055 | -0.37 | 0.21 | .075 | 0.15 | 0.21 | .470 | -0.23 | 0.21 | .262 |
| **Rum-Rum vs. Reap-Rum** | -0.32 | 0.22 | .139 | 0.06 | 0.22 | .790 | 0.03 | 0.22 | .879 | 0.20 | 0.22 | .364 |
| **Rum-Rum vs. Reap-Reap** | 0.08 | 0.21 | .704 | -0.12 | 0.21 | .553 | 0.002 | 0.21 | .991 | -0.19 | 0.21 | .356 |
| Rum-Reap vs. Reap-Rum | -0.71 | 0.21 | <.001 | 0.42 | 0.21 | .049 | -0.12 | 0.21 | .589 | 0.43 | 0.21 | .047 |
| Rum-Reap vs. Reap-Reap | -0.32 | 0.20 | .121 | 0.24 | 0.20 | .232 | -0.15 | 0.20 | .473 | 0.04 | 0.20 | .842 |
| Reap-Rum vs. Reap-Reap | 0.40 | 0.21 | .063 | -0.18 | 0.21 | .404 | -0.03 | 0.21 | .886 | -0.39 | 0.21 | .073 |

Significant effects at *p* > .05 are shaded in grey.
Rum-Rum = Rumination – Rumination condition, Rum-Reap = Rumination – Reappraisal condition, Reap-Rum = Reappraisal - Rumination condition, Reap-Reap = Reappraisal – Reappraisal condition.
