# Supplementary material for: Sequential effects of reappraisal and rumination on anger during recall of an anger-provoking event
Source: PLoS One. 2019 Jan 2;14(1):e0209029. doi: 10.1371/journal.pone.0209029 (PMC6314601; doi:10.1371/journal.pone.0209029)
Supplement: S2 Tables — This appendix is a replication of the analyses in the paper with positive affect as the dependent variable. (DOCX) [file pone.0209029.s002.docx]

# S2 Tables: Analyses with positive affect

Table S2a outlines descriptive statistics for non-anger related negative affect.

**Table s2a. Means of self-reported positive affect about the angering event for each condition, with standard deviations in parentheses**

| **Time** | **Condition** | | | |
| --- | --- | --- | --- | --- |
|  | **Rumination-Rumination** | **Rumination-Reappraisal** | **Reappraisal-Rumination** | **Reappraisal-Reappraisal** |
| **1  baseline** | 3.41 (1.23) | 3.02 (.98) | 3.17 (1.04) | 3.41 (1.05) |
| **2  post anger induction** | 2.32 (1.44) | 2.11 (1.26) | 2.05 (1.16) | 2.13 (1.24) |
| **3  post first ER phase** | 2.38 (1.25) | 2.04 (1.15) | 2.38 (1.16) | 2.45 (1.32) |
| **4  post second ER phase** | 2.41 (1.28) | 2.24 (1.19) | 2.10 (1.07) | 2.54 (1.44) |
| **5  post waiting phase** | 2.67 (1.30) | 2.45 (1.25) | 2.58 (1.19) | 2.79 (1.44) |
| **6  post distrac-ting task** | 2.71 (1.27) | 2.55 (1.38) | 2.21 (1.46) | 2.48 (1.18) |

NA = non-anger related negative affect ratings; PA = positive affect ratings, ER = emotion regulation.

These analyses are exact replications of those included in the paper, but with positive affect (PA) as the dependent variable instead of anger. Please refer to the paper for a full explanation of how these analyses were conducted.

## Effect of the anger manipulation

## The results of the analyses are in Table S2b, and reveal that the manipulation also impacted PA.

**Table S2b. Comparing PA at Time 1 to PA at each of the following time-points.**

|  | **γ** | **SE** | ***p*** |
| --- | --- | --- | --- |
| **Intercept** | 3.26 | 0.10 | <.001 |
| **Time 2** | -1.10 | 0.08 | <.001 |
| **Time 3** | -0.95 | 0.08 | <.001 |
| **Time 4** | -0.93 | 0.08 | <.001 |
| **Time 5** | -0.63 | 0.08 | <.001 |
| **Time 6** | -0.76 | 0.08 | <.001 |

Time 1 (prior to the anger induction) is the reference category in these analyses, and each time-point is being compared to Time 1.

**Effects of the first reappraisal and rumination manipulation**

We found a significant interaction between Time 2 and 3 and emotion regulation strategy on PA (γ = 0.34, SE = 0.15, *p* = .028). In the rumination group, there was no difference in PA between Time 2 (*M* = 2.21) and Time 3 (*M* = 2.20; γ =-0.01, SE = 0.11, *p* = .930). In the reappraisal group, we found that there was a significant increase in PA between Time 2 (*M* = 2.09) and Time 3 (*M* = 2.42; γ = 0.33, SE = 0.11, *p* =.003).

**Influences of strategy order**

Table S2coutlines how each of the emotion regulation strategy groups changed across time. Table S2d outlines the simple effects for the tests of differences between conditions across time.

**Table S2c. Change between the time-points on PA for each of the emotion regulation conditions**

|  | **Change from Time 2 to Time 3** | | | **Change from Time 3 to Time 4** | | | **Change from Time 4 to Time 5** | | | **Change from Time 5 to Time 6** | | |
| --- | --- | --- | --- | --- | --- | --- | --- | --- | --- | --- | --- | --- |
|  | γ | SE | *p* | γ | SE | *p* | γ | SE | *p* | γ | SE | *p* |
| **Rumination-Rumination** | 0.06 | 0.15 | .710 | 0.04 | 0.15 | .804 | 0.26 | 0.15 | .090 | 0.04 | 0.15 | .804 |
| **Rumination-Reappraisal** | -0.07 | 0.15 | .624 | 0.20 | 0.15 | .178 | 0.21 | 0.15 | .165 | 0.11 | 0.15 | .462 |
| **Reappraisal-Rumination** | 0.33 | 0.16 | .043 | -0.29 | 0.16 | .081 | 0.49 | 0.16 | .003 | -0.38 | 0.16 | .022 |
| **Reappraisal-Reappraisal** | 0.32 | 0.15 | .031 | 0.09 | 0.15 | .568 | 0.26 | 0.15 | .087 | -0.32 | 0.15 | .034 |

Significant effects at *p* > .05 are shaded in grey.

**Table S2d. Tests of differences between conditions in the size of the change in PA across time-points.**

|  | Change from Time 2 to Time 3 | | | Change from Time 3 to Time 4 | | | Change from Time 4 to Time 5 | | | Change from Time 5 to Time 6 | | |
| --- | --- | --- | --- | --- | --- | --- | --- | --- | --- | --- | --- | --- |
|  | γ | SE | *p* | γ | SE | *p* | γ | SE | *p* | γ | SE | *p* |
| Rum-Rum vs. Rum-Reap | -0.13 | 0.21 | .543 | 0.16 | 0.21 | .441 | -0.05 | 0.21 | .818 | 0.07 | 0.21 | .734 |
| Rum-Rum vs. Reap-Rum | 0.27 | 0.22 | .219 | -0.32 | 0.22 | .146 | 0.23 | 0.22 | .305 | -0.41 | 0.22 | .065 |
| Rum-Rum vs. Reap-Reap | 0.27 | 0.21 | .209 | 0.05 | 0.21 | .822 | -0.0002 | 0.21 | .999 | -0.35 | 0.21 | .096 |
| Rum-Reap vs. Reap-Rum | 0.40 | 0.22 | .069 | -0.49 | 0.22 | .028 | 0.28 | 0.22 | .210 | -0.48 | 0.22 | .029 |
| Rum-Reap vs. Reap-Reap | 0.40 | 0.21 | .061 | -0.12 | 0.21 | .583 | 0.05 | 0.21 | .817 | -0.43 | 0.21 | .044 |
| Reap-Rum vs. Reap-Reap | -0.01 | 0.22 | .972 | 0.37 | 0.22 | .094 | -0.23 | 0.22 | .302 | 0.06 | 0.22 | .794 |

Significant effects at *p* > .05 are shaded in grey.
Rum-Rum = Rumination – Rumination condition, Rum-Reap = Rumination – Reappraisal condition, Reap-Rum = Reappraisal - Rumination condition, Reap-Reap = Reappraisal – Reappraisal condition.
